# *Retracted:* Retraction

**DOI:** 10.1111/jcmm.16942

**Published:** 2021-10-01

Retraction: LncRNA LINC01305 silencing inhibits cell epithelial‐mesenchymal transition in cervical cancer by inhibiting TNXB‐mediated PI3K/Akt signalling pathway. Shu‐Ping Yan, Dan‐Xia Chu, Hai‐Feng Qiu, Ya Xie, Chun‐Fang Wang, Jian‐Ying Zhang, Wen‐Cai Li and Rui‐Xia Guo. J Cell Mol Med. 2019; 23: 2656– 2666.

The above article from Journal of Cellular and Molecular Medicine, published online on 29 January 2020 on Wiley Online Library (https://doi.org/10.1111/jcmm.14161), has been retracted by agreement between the journal Editor‐in‐Chief and John Wiley & Sons Ltd. The retraction has been agreed following an investigation based on allegations raised by a third party. A detailed investigation revealed that several image elements of the experimental data were published elsewhere in a different scientific context, while duplications were found in two images in the paper. Based on the findings, the editors consider the conclusions invalid. Despite several attempts of contact with all authors of the paper, a response was not received.

